# Copy Number Amplification of the PIK3CA Gene Is Associated with Poor Prognosis in Non-lymph node metastatic Head and Neck Squamous Cell Carcinoma

**DOI:** 10.1186/1471-2407-12-416

**Published:** 2012-09-20

**Authors:** Toshihito Suda, Takanori Hama, Shu Kondo, Yuki Yuza, Mamoru Yoshikawa, Mitsuyoshi Urashima, Takakuni Kato, Hiroshi Moriyama

**Affiliations:** 1 Department of Oto-Rhino-Laryngology, Jikei University School of Medicine, 3-25-8 Nishi-shimbashi, Minato-ku, Tokyo, 105-8461, Japan; 2 Division of Molecular Epidemiology, Jikei University School of Medicine, Minato-ku, Tokyo, Japan; 3 Division of Invertebrate Genetics, National Institute of Genetics, Mishima, Japan; 4 Department of Pediatrics, Jikei University School of Medicine, Minato-ku, Tokyo, Japan

**Keywords:** PIK3CA, KRAS, BRAF, Copy number analysis, Prognostic Factor

## Abstract

**Background:**

Deregulation of the EGFR signaling pathway is one of the most frequently observed genetic abnormalities that drives cancer development. Although mutations in the downstream components of the EGFR signaling pathway, including KRAS, BRAF and PIK3CA, have been reported in numerous cancers, extensive mutation and copy number analysis of these genes in clinical samples has not been performed for head and neck squamous cell carcinoma (HNSCC).

**Methods:**

We examined the mutations and copy number alterations of KRAS, BRAF and PIK3CA in 115 clinical specimens of HNSCC obtained from surgically treated patients.

We used DNA sequencing to detect mutations and the copy number changes were evaluated by qPCR and array comparative genomic hybridization (CGH) analysis.

**Results:**

We examined the mutations and copy number alterations of KRAS, BRAF and PIK3CA in 115 clinical specimens of HNSCC obtained from surgically treated patients. We identified 3 mutations (2.6%) in K-RAS and 3 mutations (2.6%) in PIK3CA. Copy number amplification was found in 37 cases (32.2%) for PIK3CA, 10 cases (8.7%) for K-RAS and 2 cases (1.7%) for BRAF. Kaplan-Meier survival analysis revealed that copy-number amplification of PIK3CA was markedly associated with cancer relapse in patients without lymph node metastasis. (Log-rank test, p = 0.026)

**Conclusions:**

Copy number amplification of the PIK3CA gene is associated with poor prognosis in HNSCC patients without lymph node metastasis. The PIK3CA copy number status will serve as a marker of poor prognosis in patients with HNSCC.

## Background

Progress in genomics has led to the identification of oncogenes, and genetic mutations associated with carcinogenesis have been reported for many carcinomas. Furthermore, an increase in the gene copy number due to focal amplification or chromosomal polysomy is another major mechanism of oncogene activation [1]. In addition to progress in the understanding of signaling pathways, there have been pharmaceutical advances with regard to the development of drugs that target proteins in membrane receptors and the downstream signals associated with carcinogenesis. Various trials have been performed to assess individualized drugs for the treatment of cancer that target the patients’ individual genetic makeup, and numerous agents targeting various cancer-related proteins have recently been developed.

Recent studies have shown that the over expression of epidermal growth factor receptor (EGFR) is associated with a poor prognosis in patients with head and neck squamous cell carcinoma (HNSCC) [2]. In a previous study, we analyzed the mutations and phosphorylation status of EGFR in patients with HNSCC and showed that the hyperphosphorylation of EGFR was a prognostic factor for poor survival [3]. Accordingly, the EGFR signaling pathway has attracted attention in the field of HNSCC as a promising target for molecularly targeted treatment. Indeed, cetuximab, a chimeric monoclonal antibody directed against human EGFR, has been reported to be effective in the treatment of advanced HNSCC [4].

Upon binding with a ligand, EGFR activates multiple intracellular signaling pathways, including the RAS/RAF/mitogen-activated protein kinase (MAPK) pathway and the phosphoinositide 3-kinase (PI3K)/AKT/mammalian target of rapamycin (mTOR) pathway, which transmit extracellular signals to the nucleus to control cell growth and proliferation. In many types of malignancies, such as lung cancer and colorectal cancer, mutations in the genes encoding EGFR or downstream components of the signaling pathways are highly prevalent [5,6]. Interestingly, these mutations are often associated with the effectiveness of molecularly targeted drugs. For instance, mutations in KRAS are effective predictors of colorectal cancer patients’ responses to cetuximab [7], and mutations in PIK3CA are correlated with resistance to cetuximab treatment in colon cancer cell lines [8]. Therefore, in the era of personalized cancer therapy, it will be important to know whether these mutations are present in tumors to plan effective therapeutic strategies for patients. So we determined a type of the mutation to investigate in reference to some theses about HNSCC in KRAS, BRAF, and PIK3CA [9,10].

In addition to gene mutations, copy number alterations of EGFR, KRAS, PIK3CA and other signaling mediators are also critical factors that drive cancer development and determine prognoses and the sensitivity to anticancer drugs. In NSCLC, EGFR gene copy amplification, as determined by FISH, was shown to likely be associated with poor prognosis and with improved survival upon treatment with EGFR tyrosine kinase inhibitors [11]. As for HNSCC, several reports have shown that EGFR copy number alterations are associated with a poor prognosis [12]; it is also reported that PIK3CA copy number amplification is associated with lymph node metastasis [13].

Little is known about the frequency of mutations and copy number alterations of genes other than EGFR that encode components of the RAS and PI3K pathways in HNSCC. Herein, we present a sequence and copy number analysis of the RAS, RAF and PI3K genes in a collection of 115 clinical samples.

## Methods

### Patients

This prospective cohort study was approved by the Ethics Committee for Biomedical Research of the Jikei Institutional Review Board, Jikei University School of Medicine, Tokyo, Japan. All of the patients provided written informed consent. Between September 2006 and August 2009, 115 tumors were obtained from 115 HNSCC patients who underwent surgery at the Department of Head and Neck Surgery, Jikei University Hospital. And they were consecutively submitted by surgical management. Clinical information was obtained from the clinical and surgical charts. Based on the postoperative staging, the tumor-node-metastasis (TNM) classification and stage were determined according to the 6th UICC TNM classification and stage groupings [14].

### Tumor samples

In each case, the tumor samples from the primary site and surrounding normal tissue, but not the metastatic sites, were stored at −80°C after excision. The cancer tissue was divided into two specimens: one for pathological confirmation in which the sample was composed of >70% cancer cells and the other for DNA and protein extraction.

### Mutation analysis

We used DNA sequencing to detect mutations in the clinical HNSCC specimens. Polymerase chain reaction (PCR) amplification of genomic DNA was performed, and the following genes were analyzed for mutations: KRAS (exons 1 and 2); BRAF (exon 15); and PIK3CA (exons 9 and 20). These regions included those with the most common mutations previously observed in human cancer. Table 1 shows the primers and annealing temperatures used in the PCR analysis. The sequencing of the PCR products was performed using the ABI PRISM Large Dye Terminator Cycle Sequencing Ready Reaction kit and the ABI PRISM 3700 Genetic Analyzer (PE Applied Biosystems, Foster City, CA).

**Table 1:** Primer sequences and annealing temperatures for direct sequencing

**Gene**	**Exon**	**Primer sequence**	**Tm,**
PIK3CA	9	F,5’-TTGCTTTTTCTGTAAATCATCTGTG-3’	51
		R,5’-CCACAAATATCAATTTACAACCATTG-3’	
	20	F,5’-GGTATTAACATCATTTGCTCCAA-3’	52
		R,5’-CCTATGCAATCGGTCTTTGC-3’	
KRAS	1	F,5’-CATTACGATACACGTCTGCAGTCAACTGG-3’	52
		R,5’-GTGAACATCATGGACCCTGACATACTCC-3’	
BRAF	15	F,5’-TCATAATGCTTGCTCTGATAGGA-3’	52
		R,5-GGCCAAAATTTAATCAGTGGA-3’	

F, forward primer R, reverse primer Tm, annealing temparature.

We used DNA sequencing to detect mutations in the clinical HNSCC specimens. KRAS (exons 1 and 2), BRAF (exon 15) and PIK3CA (exons 9 and 20) were analyzed for mutations.

### Analysis of copy number by quantitative PCR

The copy number changes in KRAS, BRAF and PIK3CA were evaluated by qPCR using the qPCR^TM^ MasterMix for SYBR Green, as previously reported [15]. Table 2 shows the primer and annealing temperatures used in the qPCR; the qPCR reactions for each sample and each gene were performed in triplicate. Each copy number calculation was performed using the comparative C_t_ method [16]. DNA from the normal tissue from each sample was used as the control [17]; each gene copy number in the normal tissue was set as 2, and a copy number more than 4 was considered to be a gain, as in a previous study [18].

**Table 2:** Primer sequences and annealing temperatures for copy number analysis by qPCR

**Gene**	**Primer sequence**	**Tm,**
PIK3CA	F,5’- GCAAAGGTTGGTCGGTGAA -3’	60
	R,5’- GTGATCTTTGATGTTACTCTGATGCA -3’	
KRAS	F,5’- CACCCTAGACAAGCAGCCAATA -3’	60
	R,5’- AAGCCCTGCCGCAAAAA -3’	
BRAF	F,5’- CAAGTCACCACAAAAACCTATCGT -3’	60
	R,5- AACTGACTCACCACTGTCCTCTGTT -3’	

The copy number changes in KRAS, BRAF and PIK3CA were evaluated by qPCR. DNA from the normal tissue from each sample was used as the control; each gene copy number in the normal tissue was set as 2, and a copy number more than 4 was considered to be a gain.

### Analysis of the copy number by array CGH

For the samples with copy number amplification identified by qPCR, we performed an array comparative genomic hybridization (CGH) analysis using microarray slides that contained 180,000 probes (Agilent Technologies, Santa Clara, CA, USA) [19]. We defined log2 ratio +0.5 < as gain and −0.5 > as loss and all other amounts as normal.

### Statistical analysis

The Student t-test, chi-squared test and Fisher test were used to evaluate differences in the patients’ characteristics stratified by the copy number alternation. Disease-free survival curve’s end point is defined by recurrence (local relapse, new lymph node metastasis or distant metastasis) during follow. All patients conducted observation within two month after surgery and patients were periodically (every 0.5–2 months) examined on an outpatient basis to make sure they had not relapsed. Examinations consisted of standard tests, including endoscopy and computed tomography of the chest and neck. It was defined recurrence when a carcinoma was detected again in a primary tumor and cervical lymph node or distant metastases were observed on computed tomography. Over-all survival and disease-free survival curves were generated using the Kaplan-Meier method and compared using log-rank tests. Cox proportional hazard models were fitted for multivariate analysis adjusting for age, gender, smoking status, mutation status of KRAS and PIK3CA, and amplification status. Adjusted hazard ratios (HR) and 95% confidence intervals (CI) were computed. All of the statistical analyses were performed using STATA 9.1 (STATA Corp., College Station, TX). A p value < 0.05 was considered statistically significant.

## Results

### Patients’ characteristics

The patients ranged in age from 32 to 88 years, and there were more men than women in the cohort. Most of the patients had oropharyngeal or oral cavity cancer, and 47% had stage IV disease. The patient characteristics are listed in (Table 3).

**Table 3:** Patients’ characteristics

**Variable**	**Total (n = 115)**
Age : mean ± SD	65.1 ± 11.0
Sex: Male/female	93/22
Primary site	
Oropharyngeal	25(21.7%)
Hypopharyngeal	25(21.7%)
Laryngeal	23(20%)
Oral cavity	31(27%)
Nasal cavity	11(9.6%)
Tumor grade:	45(39.1%)/48(41.7%)/22(19.1%)
well/ moderate/poor	
T stage: T1/T2/T3/T4	16/45/27/27
	(13.9%/39.1%/23.5%/23.5%)
N stage: N0/N1/N2/N3	59/17/39/0
	(51.3%/14.8%/33.9%/0%)
Stage: I/II/III/IV	12/23/26/54
	(10.4%/20%/22.6%/47.0%)

115 patients were obtained in this study. The patient characteristics are listed.

### Mutation analysis of KRAS, BRAF and PIK3CA

We conducted a sequencing analysis to examine the frequency of mutations in the KRAS, BRAF and PIK3CA genes, and we investigated the correlations between the presence of mutations and the clinical data. Specifically, we sequenced KRAS exon 1and exon2, BRAF exon 15 and PIK3CA exon 9 and exon 20 and identified mutations in 6 of the 115 patients (5.2%). Three mutations were observed in exon 1 of KRAS, and all were the same substitution (G12A). No mutation was found in exon 2.The primary tumors included two oral cavity carcinomas and one oropharyngeal carcinoma; in terms of lifetime smoking status (pack-years), one patient had a high score (more than 30 pack-years), and the others had low scores [20]. Three mutations in PIK3CA were found: two in exon 9 and one in exon 20, and all of the mutations were substitutions (E545K and H1047R). The primary tumors were two oral cavity carcinomas and one laryngeal carcinoma, and all three patients were non-smokers. No BRAF mutations were detected (Table 4). Significant association was absent about the association between these mutations and clinical data (localization, sex, age,stage etc.).

**Table 4:** HNSCC gene mutations

**No**	**Nucleotide change**	**Amino acid Change**	**Primary tumor**	**Exon**	**Smoking (pack/year)**	**Age/Sex**
12	Substitution of G for C at nucleotide 35	G12A	Oral Cavity (T3N0M0)	KRAS Exon1	45	51/M
21	Substitution of G for C at nucleotide 35	G12A	Oropharyngeal (T2N0M0)	KRAS Exon1	10	47 /M
30	Substitution of G for C at nucleotide 35	G12A	Oral Cavity (T3N1M0)	KRAS Exon1	0	54/M
10	Substitution of A for G at nucleotide 3140	H1047R	Laryngeal (T3N0M0)	PIK3CA Exon20	0	61/M
51	Substitution of G for A at nucleotide 1633	E545K	Oral Cavity (T1N1M0)	PIK3CA Exon9	0	79/M
53	Substitution of G for A at nucleotide 1633	E545K	Oral Cavity (T3N0M0)	PIK3CA Exon9	0	82/M

We sequenced KRAS exon 1, BRAF exon 15 and PIK3CA exon 9 and exon 20 and identified mutations in six of the 115 patients (5.2%).

### Copy number analysis of KRAS, BRAF and PIK3CA

We identified copy number changes in PIK3CA, K-RAS and BRAF. PIK3CA copy number amplification was found in 37 cases (32.2%), whereas only 10 cases (8.7%) were found for K-RAS and 2 cases (1.7%) for BRAF (Table 5). In PIK3CA amplified cases, one case had PIK3CA mutation, 2 cases had KRAS mutation and no case had both mutations. The patients’ characteristics stratified by the PIK3CA copy number alteration are shown in (Table 6); there was no significant association. As with PIK3CA, no difference was observed for the KRAS and BRAF copy number alternation (data not shown).

**Table 5:** Copy number alteration of K-RAS, PI3CA, BRAF

	**Not Amplified**	**Amplification**	**Deletion**
K-RAS (12p12.1)	103(89.6%)	10(8.7%)	2(1.7%)
PIK3CA(3q26.3)	77(67.0%)	37(32.1%)	1(0.9%)
BRAF (7q34)	106(92.2%)	2(1.7%)	7(6.1%)

We identified copy number changes in PIK3CA, K-RAS and BRAF. PIK3CA copy number amplification was found in 37 cases (32.2%).

**Table 6:** Patients’ characteristics stratified by Copy number alternation of PIC3CA

**Variable**	**Copy number of PIK3CA (Not Amplified n = 78)**	**Copy number of PIK3CA (Amplification n = 37)**	***P *value**
Age: mean ± SD	64.8 ± 11.6	65.6 ± 9.9	0.60^*^
Sex: Male/Female	63 / 15	30 / 7	0.96^†^
Number of metastatic			
Lymph nodes:	0/0/1.5	0/0/0	0.65^‡^
25%/50%/75%			
Primary site			0.39^†^
Oropharyngeal	15(19.2%)	10(27.0%)	
Hypopharyngeal	16(20.5%)	9(24.3%)	
Laryngeal	15(19.2%)	8(21.6%)	
Oral cavity	22(28.2%)	9(24.3%)	
Nasal cavity	10(12.8%)	1(2.7%)	
Tumor grade:	28/35/15	18/12/7	0.37^†^
well/ moderate/poor	(35.9%/44.9%/19.2%)	(48.6%/32.4%/18.9%)	
TNM classification			
T stage: T1/T2/T3/T4	12/30/17/19	4/15/10/8	0.85^†^
	(15.3%/38.5%/21.8%/24.4%)	(10.8%/40.5%/ 27.0%/21.6%)	
N stage: N0/N1/N2	43/12/23	16/5/16	0.34^†^
	(55.1%/15.4%/29.5%)	(43.2%/13.5%/43.2%)	
Stage : I/ II/III/IV	11/16/17/34	1/7/9/20	0.28^†^
	(14.1%/20.5%/21.8%/43.6%)	(2.7%/18.9%/ 24.3%/54.1%)	
Pack-year(tobacco) mean ± SD	19.6 ± 24.3	22.3 ± 24.6	0.64^*^

^*^ Student t test † Chi-square test ‡ Fisher test.

The patients’ characteristics stratified by the PIK3CA copy number alteration. There was no significant association.

### Kaplan-Meier curves of over-all survival and disease-free survival by Copy number status

**Figure 1:**
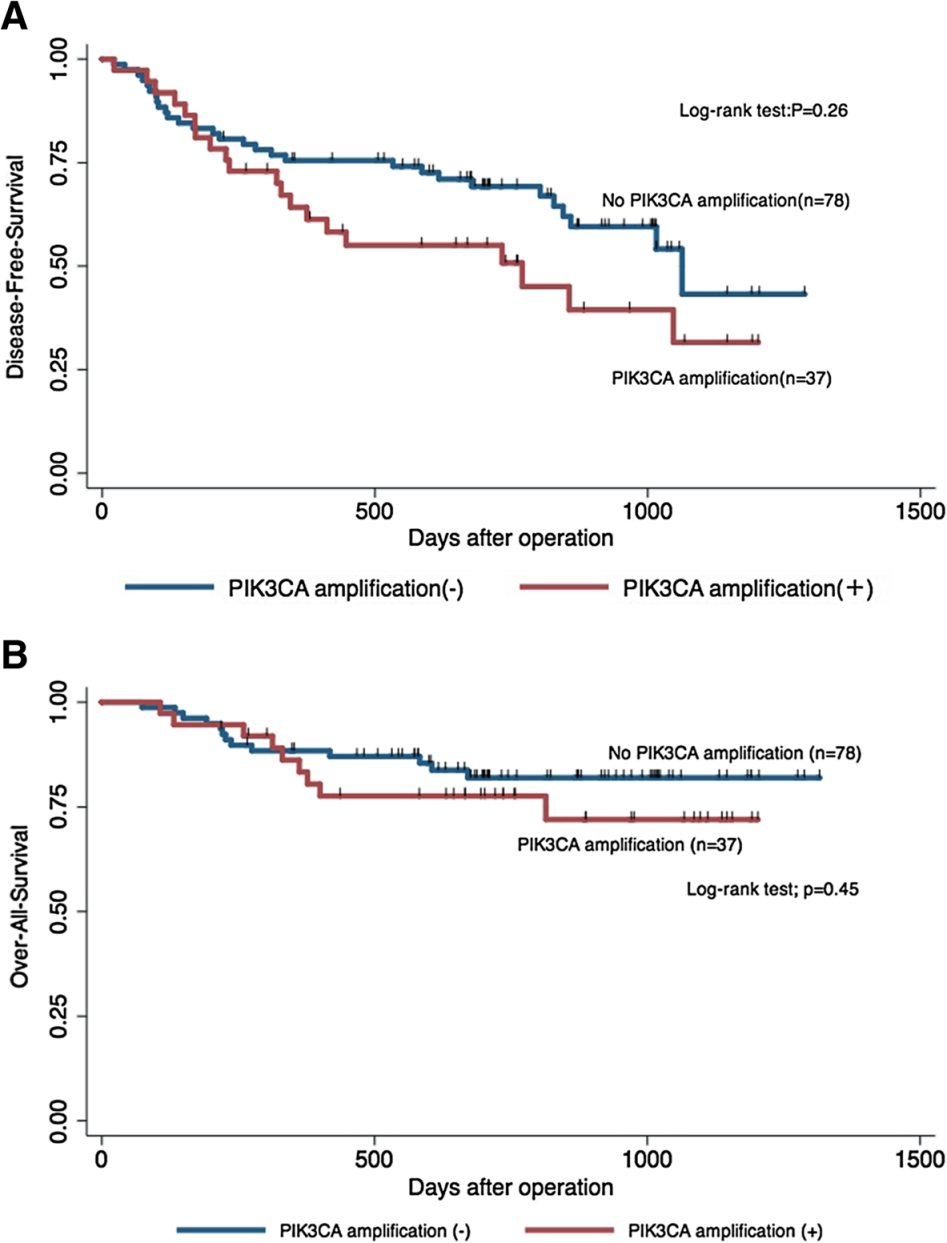
**115 patients with PIK3CA copy number status.** Kaplan-Meier curves for disease-free survival (**A**) and over-all survival (**B**). Patients were with or without PIK3CA copy number amplification. The p-values were estimated using the log-rank test

Tumor recurrence and death occurred in 49 patients (42.6%) and 22 patients (19.1%), respectively during the median follow-up period of 723 days. Over-all survival and disease-free survival curves were generated using the Kaplan-Meier method, and log-rank tests were used to determine if survival was associated with the copy number status of PIK3CA. There was no significant association between the copy number status (with or without amplification) in PIK3CA and the over-all survival and disease-free survival (Log-rank test, p = 0.45 and p = 0.26) (Figure 1). We conducted same examination in KRAS copy number status, and the significant difference was not found in the copy number status and prognosis.

**Figure 2:**
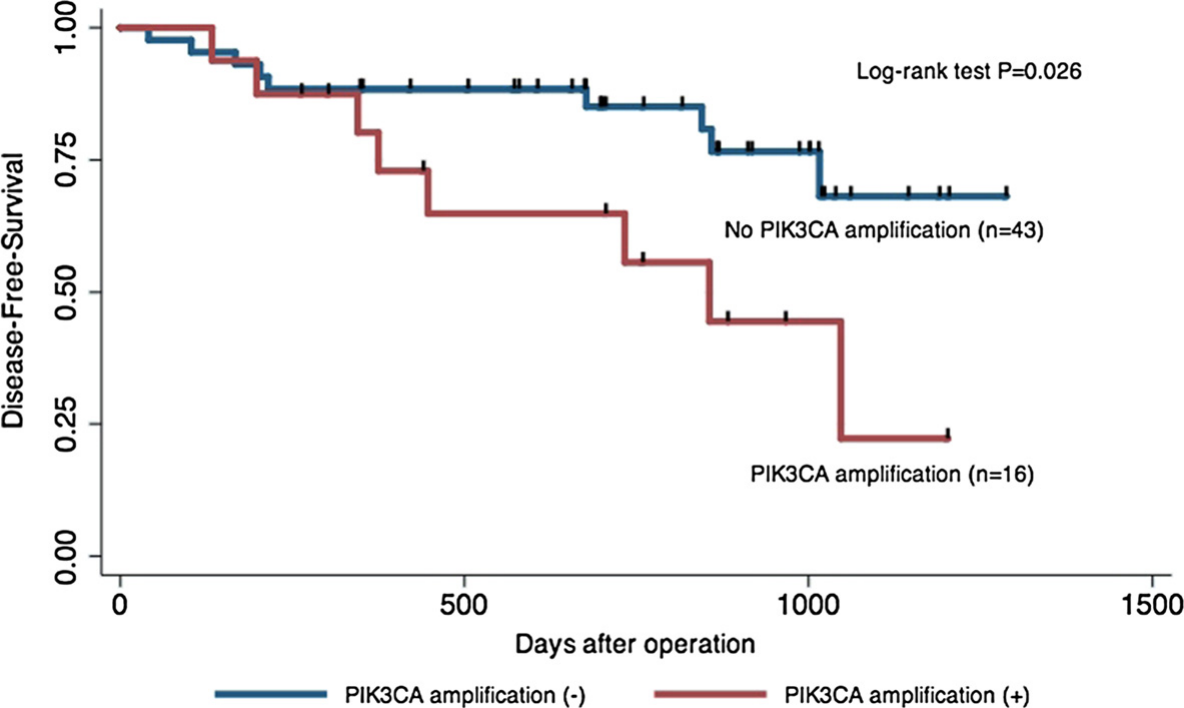
**Restricting 59 patients without lymph node metastases.** Kaplan-Meier curves for disease-free survival. Patients with or without amplification in PIK3CA, restricting to 59 patients without lymph node metastases. The p-values were estimated using the log-rank test

However, after excluding the patients with lymph node metastasis, the disease-free survival curves drawn using the Kaplan-Meier method about 59 patients without lymph node metastasis (N stage = N0 patients) indicated that the patients with PIK3CA copy number amplification (n = 16) showed earlier recurrence than those without PIK3CA copy number amplification (n = 43) (log-rank test, *p* =0.026) (Figure 2). Of those with PIK3CA copy number amplification, 31% were disease-free at 2.0 years, whereas 90% of the patients without PIK3CA copy number amplification survived without recurrence during the study period. Actually 19 patients had a recurrence in PIK3CA amplified patients and 30 patients had a recurrence in not amplified patients. Copy number amplification of the PIK3CA is associated with poor prognosis in HNSCC patients without lymph node metastasis.

### Cox proportional hazard models with multivariate adjustment

Cox proportional hazard models with disease-free survival were computed to determine the significance of PIK3CA amplification with adjustment for age, gender, smoking status and mutation status (Table 7). Without multivariate analysis, PIK3CA amplification showed a significant crude HR. With multivariate analysis, patients with PIK3CA amplification showed poor prognostic markers: adjusted HR, 3.13; 95% CI, 1.16 to 8.40; P =0.024. In contrast, the factor (age, gender, smoking status and mutation status) showed no significant association with disease-free survival.

**Table 7:** Cox proportional hazard models (59 patients without lymph node metastasis)

**Variable**	**Crude HR**	**95% CI**	***P *value**	**AHR**	**95% CI**	***P *value**
PIK3CA amplification	2.83	1.08-7.36	0.003	3.13	1.16-8.4	0.024
Mutation (PIK3CA K-Ras)	0.73	0.09-5.6	0.77	0.83	0.1-6.78	0.86
Age	0.97	0.93-1.00	0.13	0.95	0.91-1.0	0.075
Gender	1.22	0.52-2.84	0.64	1.06	0.42-2.65	0.89
Smoking status	1.01	0.99-1.03	0.08	1.02	0.99-1.04	0.06

Adjusted for age, gender, smoking status, mutation status.

HR, hazard ratio; CI, confidence interval; AHR, adjusted hazard ratio.

Cox proportional hazard models were computed to determine the significance of PIK3CA amplification with adjustment for age, gender, smoking status and mutation status.

## Discussion

The overall mutation frequency of KRAS, BRAF and PIK3CA in our HNSCC samples was lower than reported for other cancers. We identified mutations in 2.6% of our samples for the KRAS gene, 2.6% for PIK3CA and 0% for BRAF. KRAS mutations are observed in approximately 45-60% of pancreatic cancers [21,22], 30-50% of colorectal cancers [23,24] and 30% of non-small-cell lung carcinomas [25]. BRAF mutations are observed in approximately 60-70% of malignant melanomas [26,27], 40% of thyroid carcinomas [28] and 5-10% of colorectal cancers [29]. PIK3CA mutations are observed in 32% of colorectal cancers [30], 31% of endometrial cancers [31] and 14% of breast cancers [32]. The small sample size may have caused less frequency of these mutations in the study.

The KRAS mutations identified in three of our tumor samples (2.6%) were identical G12A substitutions, which is a well-characterized activating mutation. The low KRAS mutation frequency in the present study is in agreement with previous small-scale studies of HNSCC that reported a mutation frequency of 2.4% (1/42 samples) in oral squamous cell carcinoma [10], 4.5% (1/22 samples) in oropharyngeal cancer [33] and 0% (0/16 samples) in HNSCC [9]. We also found that the frequency of copy number amplification was not high (8.7%), suggesting that approximately 90% of the patients have normal KRAS proteins and that the hyper activation of KRAS, per se, is not a common feature of HNSCC. A recent study showed that KRAS mutations result in resistance to cetuximab in colorectal cancer, limiting the utility of this drug [34]. In lung cancer, although the frequency of KRAS mutations is similar to that in colorectal cancer, these mutations are not reported to be a predictive marker. Therefore, the low KRAS mutation frequency of HNSCC may cause difficulty in predicting the effect of cetuximab and other EGFR inhibitors.

We analyzed exon 15 of BRAF to search for a V600E substitution, the most common activating mutation of BRAF, which is observed at high frequencies in various cancers [35]. However, we did not detect any V600E mutations in our samples, and the frequency of copy number alteration was also low, at 1.7% (2/115). Together with a previous study that reported a BRAF mutation frequency of 2.4% (1/42) in oral squamous cell carcinoma [10], our results suggest that the BRAF mutation frequency is much lower in HNSCC than in other cancers.

Mutations in exons 9 and 20 of PIK3CA were found in three specimens (2.6%), and these mutations are known to be hotspot mutations that generate a constitutively active kinase in other cancers [36]. The mutation frequency of PIK3CA in our study was somewhat lower than that reported in previous small-scale studies. One study of surgical specimens from 30 Americans with HNSCC and eight HNSCC cell lines found mutations in four out of the 38 samples (10.5%) [37]. Another study found mutations in two surgical specimens and five cell lines among 54 cases, including 17 cell lines and 18 surgical specimens from Vietnamese patients and 19 surgical specimens from Indian patients [38]. The lower mutation frequency may reflect differences in the genetic backgrounds or may be due to the small population sizes. Although the frequency of point mutations in PIK3CA was low, we found copy number amplification in 37 samples (32.2%), suggesting that the hyper activation of the PI3K pathway occurs in one-third of HNSCC patients. PIK3CA mutations are associated with resistance to cetuximab in colorectal cancer in vitro [8]. Furthermore, a phase I study of a recently developed mTOR inhibitor found that colorectal cancer patients with PIK3CA mutations had a higher response rate than those without mutations [39]. Thus, checking the status of the PIK3CA gene will be important when using these molecularly targeted drugs in HNSCC patients. However, the low frequency of mutation in PIK3CA complicates its utility as a predictive marker for use in molecularly targeted medicine in clinical settings.

In our analysis, an amplification of the PIK3CA copy number was found in many of the samples (32.2%). With regard to lung cancer, such amplification is more frequent in squamous cell carcinomas (33.1%) than adenocarcinomas (6.2%) [40]. Interestingly, Fendri et al. reported that the frequency of amplification was 21.6% and was associated with lymph node metastasis and with the overall survival in nasopharyngeal cancer [41]. In HNSCC, metastatic lymph nodes are very strongly associated with disease progression and clinical staging: the cancer is considered stage III or IV when lymph node metastases are found. Therefore, we hypothesized that the copy number amplification of PIK3CA was associated with a poor prognosis. The frequency of the copy number alteration observed in the present study was the same as in other reports, yet we found no significant difference in the disease-free survival and overall survival between patients with PIK3CA amplification and those without amplification. However, when the patients with lymph node metastases were excluded from the population, a significant correlation was found between PIK3CA copy amplification and the time to relapse (log-rank test, *p* =0.026). As cancer relapse occurs in the lymph nodes in most cases, the amplification of the PIK3CA copy number is likely to promote the process of lymph node metastasis in early-stage patients. Our evidence further suggests that those patients with early-stage HNSCC may be divided into two subgroups of good and poor prognoses, as defined by the copy number status of PIK3CA.

The limitations of this study include the small number of patients especially about the prognosis study by the status of the copy number and may be include the selection bias in undergoing surgery.

## Conclusions

We examined the mutations and copy number alterations of KRAS, BRAF and PIK3CA in 115 clinical specimens of HNSCC. We identified 3 mutations (2.6%) in K-RAS and 3 mutations (2.6%) in PIK3CA. Copy number amplification was found in 37 cases (32.1%) for PIK3CA, 10 cases (8.7%) for K-RAS and 2 cases (1.7%) for BRAF. Kaplan-Meier survival analysis revealed that copy-number amplification of PIK3CA was markedly associated with cancer relapse in patients without lymph node metastasis. (Log-rank test, p = 0.026) Copy number amplification of the PIK3CA gene is associated with poor prognosis in HNSCC patients without lymph node metastasis.

## Competing interests

No potential conflicts of interest were disclosed.

## Authors’ contributions

Conception/Design: TH, TS, MU Financial support: TH, YY, MU Administrative support: TH, TS, SK, YY, MU Provision of study materials: TH, TS, TK, HM Collection/assembly of data: TH, TS, MY, TK, HM, Data analysis: TH, TS, MU Manuscript writing: TH, TS, SK. All authors read and approved the final manuscript.

## Pre-publication history

The pre-publication history for this paper can be accessed here:

http://www.biomedcentral.com/1471-2407/12/416/prepub
